# Implementation of transcutaneous ultrasound-guided axillary vein access for implantations, revisions and upgrades of cardiac implantable electronic devices in a large tertiary care center

**DOI:** 10.1007/s00392-025-02692-7

**Published:** 2025-06-24

**Authors:** Julius Nikorowitsch, Tahsin Üctas, Katrin Borof, Andreas Metzner, Jan-Per Wenzel, Simon Julius Winkelmann, Simon Pecha, Yalin Yildirim, Hermann Reichenspurner, Paulus Kirchhof, Tobias Tönnis, Nina Becher

**Affiliations:** 1 Department of Cardiology, University Heart & Vascular Center Hamburg, University Medical Center Hamburg-Eppendorf, Hamburg, Germany; 2 German Center for Cardiovascular Research (DZHK), Partner Site Hamburg/Kiel/Luebeck, Hamburg, Germany; 3 Department of Rhythmology, University Heart Center Lübeck, University Hospital Schleswig-Holstein, Ratzeburger Allee 160, 23538 Lübeck, Germany; 4 Department of Cardiovascular Surgery, University Heart & Vascular Center Hamburg, University Medical Center Hamburg-Eppendorf, Hamburg, Germany; 5 Institute of Cardiovascular Sciences, University of Birmingham, Birmingham, UK

**Keywords:** Venous access, Ultrasound-guided (US), Cardiac implantable electronic devices, Pacemaker, Device complications

## Abstract

**Background:**

Central venous access for cardiac implantable electronic device (CIED) implantations is conventionally acquired via the cephalic or subclavian vein. Controlled data suggest that axillary vein access may reduce complications.

**Objectives:**

We, therefore, shifted institutional practice from subclavian vein access to ultrasound (US)-guided axillary vein access for new implantations and revisions or upgrades and report on implant success rates, learning curves and periprocedural complications.

**Methods:**

Between January 2021 and August 2023, all patients undergoing CIED implantations, revisions or upgrades were analyzed. US-guided axillary access was introduced starting with one operator and spreading to most operators and trainees thereafter. Periprocedural outcomes and complications (pocket hematoma, hemothorax, and pneumothorax) of transcutaneous US-guided axillary vein access were compared to the subclavian vein access.

**Results:**

In this study, 986 patients (median age: 75 years, interquartile range (IQR) 64–82 years, 35% women) with 87% new implantations and 13% revisions or upgrades were included. Transcutaneous US-guided axillary access was successful in 535/578 patients (93%), subclavian vein access in 400/408 patients (98%) (*p* < 0.001). For device upgrades or revisions specifically, axillary access was successful in 69/79 patients (87%), versus 45/47 patients (96%) with subclavian access (*p* = 0.208). The learning curve for axillary access was steep with success rates of 93 after 30 cases per operator. Complications occurred in 2/578 patients (0.3%) undergoing axillary vein access versus 17/408 patients (4.2%) (*p* < 0.001) undergoing subclavian vein access.

**Conclusion:**

The implementation of transcutaneous US-guided axillary vein access for implantation, revisions and upgrades of cardiac electronic devices is feasible in a large tertiary care center. The periprocedural complications are rare.

**Graphical abstract:**

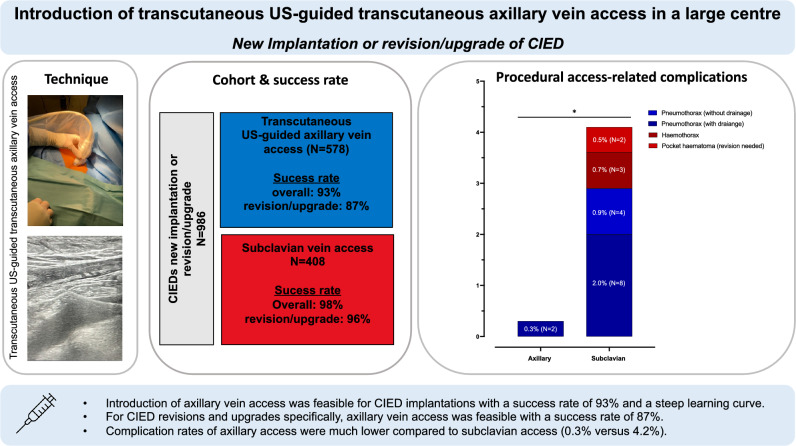
*CIED* cardiac implantable electronic device, * US* ultrasound

**Supplementary Information:**

The online version contains supplementary material available at 10.1007/s00392-025-02692-7.

## Introduction

Central venous access is required to implant, revise or upgrade pacemakers, implantable cardioverter defibrillators (ICD), and cardiac resynchronization therapy (CRT). These operations are performed in approximately 1.3 million patients per year [1]. Vascular access is typically gained via the subclavian (40% in 2013 in Europe), or cephalic vein (60% in 2013) [2]. Cephalic vein access is limited by lower success rates [3, 4], while subclavian access is associated with periprocedural pneumothorax varying between 1.2% [5] and 2.4% [6]. In addition, subclavian access can lead to subclavian crush during long-term follow-up [5, 7]. As a result, its recommendation was downgraded to a secondary access option in a recent consensus document on CIED implantation techniques [8].

To reduce periprocedural complications, the operators sought other ways to gain central venous access. Fluoroscopy-guided access to the axillary vein for cardiac implantable electronic devices (CIED) implantation was first described in 1997 and refined thereafter [9, 10]. The conceptual advantages of axillary vein access include easier access and a large vessel diameter suitable for multiple leads [3, 11, 12].

Fluoroscopy-guided axillary vein access yielded higher success rates than cephalic vein access in three small randomized trials [3, 10, 11]. These findings are underpinned by a meta-analysis which additionally demonstrates the superiority of fluoroscopy-guided axillary vein access over subclavian vein access [13]. Intra-pocket ultrasound (US) can guide axillary vein access without radiation exposure. This method led to higher success rates, shorter procedure times, and reduced radiation exposure compared to cephalic vein access in the ACCESS trial (intra-pocket ultrasound-(US) guided axillary vein access versus cephalic vein access for implantation of cardiac electronic devices) and other small trials [14, 15]. Recently, the fluoroscopic approach was compared to US guided axillary vein access in the ZEROFLUOROAXI (ultrasound guided axillary access vs standard fluoroscopic technique for cardiac lead implantation) trial [16]. The success rates and complications were the same but the US-guided access reduced radiation exposure in line with results from a retrospective analysis [17]. Beforehand, three small studies reported the feasibility of US-guided axillary vein puncture for new CIED implantations including only up to 70 patients each [18–20]. These data encouraged the device team at our center to systematically introduce US-guided axillary access for all device procedures in a standardized way. Here, we report the feasibility, learning curves and periprocedural clinical outcomes of almost 1000 patients that were operated during the gradual switch from subclavian access to transcutaneous US-guided axillary vein access for new CIED implantations and revisions or upgrades.

## Methods

### Study design

#### Study population

**Fig. 1:**
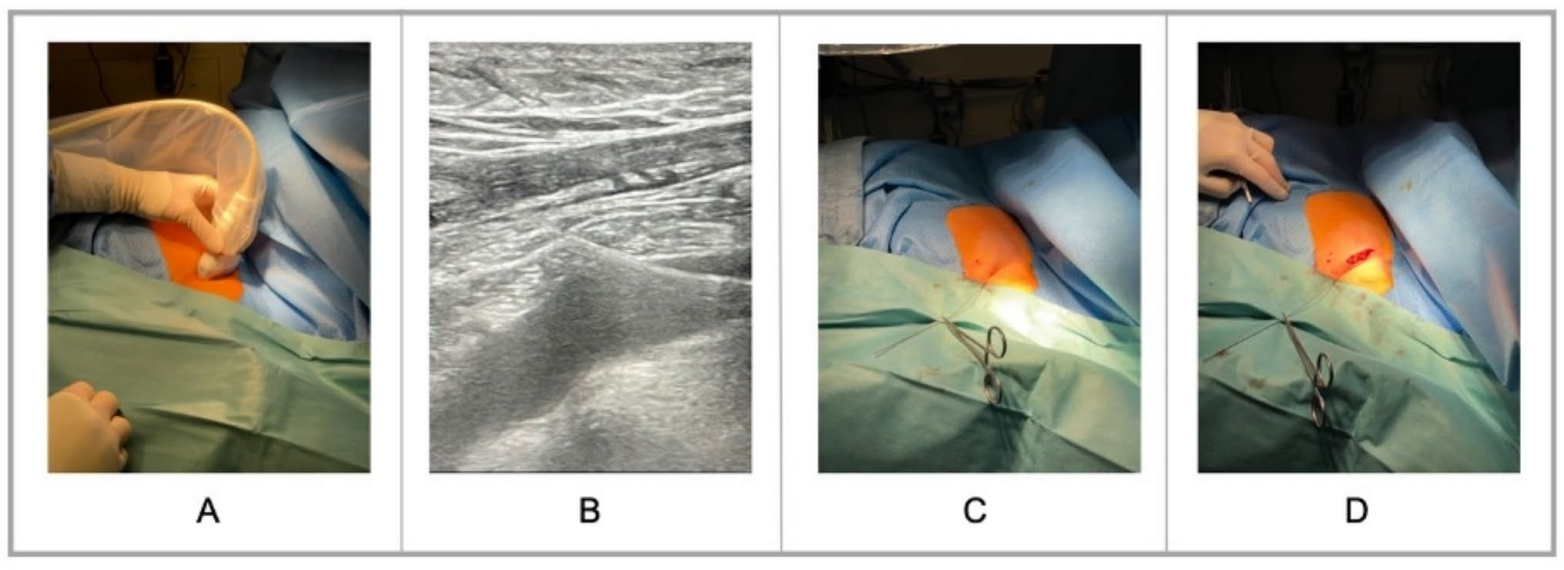
Technique of the transcutaneous US-guided axillary vein access **A** Linear array US-probe (11L) with a sterile cover placed at the deltopectoral groove**. B** After puncture of the axillary vein using the in-plane technique a guidewire is introduced **C** Transcutaneous wire introduction** D** After dissecting the subcutaneous tissue the wire(s) were threaded through the incision followed by standard lead and generator implantation

All consecutive patients undergoing CIED implantation, revision or upgrade at the University Medical Center Hamburg–Eppendorf in Germany between January 2021 and August 2023 were retrospectively included in the analysis (Fig. 1). Of the 1020 patients, 14 patients undergoing cephalic vein and 20 patients undergoing fluoroscopic axillary vein access (*N* = 20, 2%) were excluded. After exclusions, 968 patients remained for analysis.

Eligible patients underwent new implantations of single, dual or three-chamber pacemakers or defibrillators. The revisions were performed upon malfunction or dislocation of any CIED lead. The patients were upgraded to ICDs or with additional leads as appropriate. Indications were according to current guidelines [21].

The project was approved by local Ethics committee (2023–300302-WF, State of Hamburg Chamber of Medical Practitioners). Individual patient consent to the analysis of anonymized data was waived, in line with local regulations (§12 HmbKHG) and with the Declaration of Helsinki.

#### Operators

All procedures were performed in a tertiary care center. JN switched to axillary access during his second year of training for CIED implantations, based on data from Tagliari et al. [20]. Subsequently, further operators switched from the institutional standard of subclavian access to US-guided axillary vein access. The operations were performed by highly experienced operators (> 10,000 CIED procedures) and beginners. Fellows and trainees were routinely supervised by an experienced operator. Venous access was chosen at the discretion of the operator.

#### Transcutaneous, US-guided axillary venous access

Following complete antiseptic precautions and local anesthesia, a linear array US probe (11L) with a sterile cover was placed at the deltopectoral groove (Fig. 1A). Sedation or general anesthesia, in addition to local anesthesia, was administered only to patients receiving an ICD generator, a left ventricular lead, or to those unable to tolerate the procedure due to pain or anxiety. The axillary vein was distinguished from the axillary artery by compression, its more caudal location or color Doppler imaging. The US probe was then placed in line with the axillary vein (in-plane technique). A standard 18G needle without special US visibility, which came with the standard peelable sheaths from different manufacturers, was used for the venous puncture. The needle attached to a syringe filled with saline was advanced through the skin towards the axillary vein while visualizing the needle tip. If blood was aspirated, the 0.035″ J guidewire was advanced through the needle using the Seldinger technique (Fig. 1B and C). The venous puncture was performed with the patient in a prone position, without a head-down tilt. The fluoroscopic localization of the wire in the superior vena cava confirmed correct central venous access. The procedure was repeated according to the number of leads planned to be implanted. This approach was adapted for all pacemaker and ICD leads including right atrial as well as right and left ventricular leads. An adjacent skin incision approximately one centimeter caudally parallel to the deltopectoral groove was created. After dissecting the subcutaneous tissue, the wire(s) were threaded through the incision followed by sheath insertion via the guidewire and standard lead and generator implantation (Fig. 1D).

#### Subclavian venous access

Subclavian access was the standard central venous access procedure at the start of the study. The anatomical landmark-guided subclavian vein access was achieved through the intra-thoracic method described before [8]. Fluoroscopy or venograms were only used if the first attempt of the landmark-guided approach failed.

#### Statistical analysis and outcomes

All data were collected retrospectively through the review of patient records. Continuous parameters are reported as median and interquartile range (IQR) and categorical parameters as frequencies and percentages. For between group comparisons, Wilcoxon rank sum test, Pearson’s Chi-squared test and Fisher’s exact test were used. Patients were grouped according to the first intended venous access site. Periprocedural access-related complications included pocket hematoma requiring revision, hemothorax, and pneumothorax, either managed conservatively or requiring drainage. The follow-up period included the inpatient stay, which consisted of at least one overnight stay.

To characterize the learning process to perform a successful axillary vein access the percentage of success was calculated in the 1st to 10th, 11th to 30th, 31st to 50th and all following (> 50th) cases by operators with at least 30 operations. Furthermore, for all cases of these six operators, a learning curve for successful use of the axillary access was calculated from cubic splines from univariate logistic regression models using MLE based on the number of procedures performed.

Univariate and multivariate logistic regression analyses were performed to identify predictors of US-guided axillary access failure. A two-sided p-value < 0.05 was considered significant. Statistical analyses were performed in R version 4.1.2.

## Results

### Patient and procedural characteristics

**Fig. 2:**
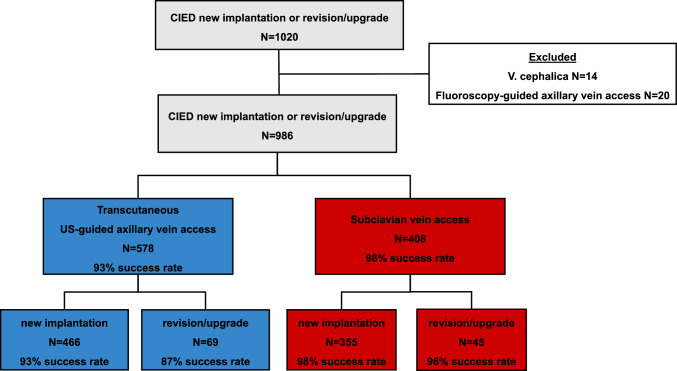
PRISMA diagram of the study cohort. *CIED* cardiac implantable electronic device, *US* ultrasound

**Fig. 3:**
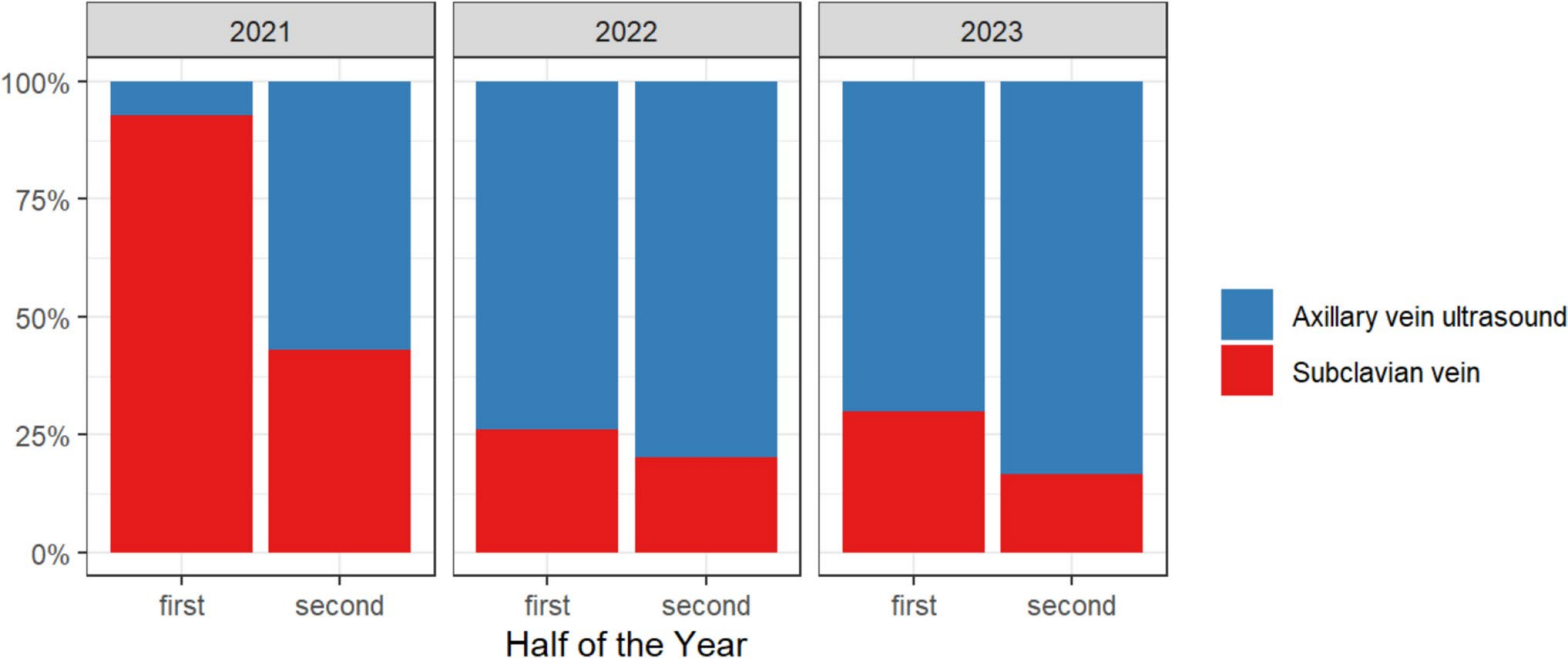
Number of procedures per half a year stratified according to transcutaneous US-guided axillary vein access (blue) and subclavian vein access (red) as first puncture site

Between January 2021 and August 2023, 986 patients (median age 75 years, interquartile range (IQR): 64–82), 349 women (35%)) underwent implantation of a CIED (Table 1, Fig. 2 and Supplement Table S1). New implantations were performed in 860 patients (87%), upgrades or revisions in 126 patients (13%). A total of 1636 new leads were implanted. In 578/986 patients (59%) axillary, US-guided access was used. Subclavian vein access was chosen in 408/986 patients (41%) as the primary puncture site. The baseline characteristics did not differ between patients undergoing CIED implantation via axillary, US-guided access versus subclavian vein access except for more patients with diabetes, prior thoracic surgery, coronary artery disease and antithrombotic therapy in those with a subclavian vein access (Table 1). The majority of procedures (592/986 operations, 62%) was performed by four experienced operators. Twenty-four operators contributed each less than 31 operations (197/986 operations, 20%) (Supplement Table S2). After starting with high rates of subclavian access, US-guided axillary vein access was increasingly adopted during the study period (Fig. 3). The procedure time was longer in patients undergoing axillary vein access (median 70 min (IQR 54–98) vs. 62 min (IQR 49–81, *p* < 0.001) but decreased over the study period (Supplement Figure S1). Fluoroscopy time did not differ (*p* = 0.065). Further procedural details are shown in Table 2.

**Table 1:** Baseline characteristics according to vein access (transcutaneous US-guided axillary vein access vs. subclavian vein access) for new implantation and revision and upgrades of CIED

	Transcutaneous US-guided axillary vein access (*N* = 578)	Subclavian vein access (*N* = 408)	Total (*N* = 986)	p-value
Demographic characteristics				
Age, [years] (IQR)	74 (63, 82)	75 (65, 82)	74 (64, 82)	0.349
Women, *N* (%)	216 (37)	133 (33)	349 (35)	0.123
BMI [kg/m^2^]	26.0 (23.2, 30.0)	26.0 (23.3, 29.2)	26.0 (23.2, 29.6)	0.593
Comorbidities, *N* (%)				
Hypertension	346 (60)	258 (63)	604 (61)	0.284
Diabetes	117 (20)	108 (26)	225 (23)	0.022
Current and prior smoking	132 (23)	105 (26)	237 (24)	0.294
Coronary artery disease	250 (43)	211 (52)	461 (47)	0.009
Prior thoracic surgery	16 (2.8)	27 (6.6)	43 (4.4)	0.004
HFrEF	202 (36)	123 (30)	325 (33)	
HFmREF	55 (9.7)	49 (12)	104 (11)	
HFpEF	28 (4.9)	21 (5.2)	49 (5.0)	
LVEF [%]	50 (33, 60)	54 (35, 60)	52 (35, 60)	0.086
Pulmonary disease	83 (14)	46 (11)	129 (13)	0.157
COPD	45 (7.8)	21 (5.1)	66 (6.7)	0.103
Antiplatelet therapy/anticoagulation, *N* (%)				
No	356 (62)	218 (53)	574 (58)	0.005
ASS	130 (22)	106 (26)	236 (24)	
Clopidogrel	54 (9.3)	33 (8.1)	87 (8.8)	
DAPT	37 (6.4	48 (12)	85 (8.6)	
Ticagrelor/Prasugrel	1 (0.2)	3 (0.7)	4 (0.4)	
Device indication, *N* (%)				
AV block	248 (43)	247 (61)	495 (50)	
Sinus node dysfunction	127 (22)	63 (15)	190 (19)	
Binodal disease	0 (0)	1 (0.2)	1 (0.1)	
Slow AF	38 (6.6)	29 (7.1)	67 (6.8)	
Resynchronization therapy	45 (7.8)	16 (3.9)	61 (6.2)	
ICD for primary prevention	92 (16)	33 (8.1)	125 (13)	
ICD for secondary prevention	170 (17)	116 (20)	54 (13)	
Type of device, (*N* %)				
Single chamber PM	84 (8.5)	51 (8.8)	33 (8.1)	
Dual chamber PM	294 (51)	277 (68)	571 (58)	
Single chamber ICD	97 (17)	42 (10)	139 (14)	
Duale chamber ICD	38 (6.6)	60 (6.1)	22 (5.4)	
CRT-P	43 (4.4)	31 (5.4)	12 (2.9)	
CRT-D	89 (9.0)	67 (12)	22 (5.4)	
Number of new leads, *N* (%)				
0 (old lead used)	2 (0.3)	1 (0.2)	3 (0.3)	< 0.001
1	197 (34)	105 (26)	302 (31)	
2	318 (55)	287 (70)	605 (61)	
3	61 (11)	15 (3.7)	76 (7.7)	

Data are median (IQR) or number (%)

*AF* atrial fibrillation, *ASS* acetylsalicylic acid, *BMI* body mass index, *DAPT* dual antiplatelet therapy, *CIED* cardiac implantable electronic devices, *COPD* chronic obstructive pulmonary disease, *CRT-D/-P* cardiac resynchronization therapy defibrillator or pacemaker, *LVEF* left ventricular ejection fraction, *HFmreF* heart failure with mildly reduced ejection fraction, *HFpEF* heart failure with preserved ejection fraction, *HFreF* heart failure with reduced ejection fraction, *ICD* implantable cardioverter defibrillator, *US* ultrasound, *PM* pacemaker

**Table 2:** Outcomes of the study. Procedural success and perioperative complications by venous access group (transcutaneous US-guided axillary vein access vs. subclavian vein access) for new implantation and revision and upgrades of CIED

	Transcutaneous US-guided transaxillary vein access (*N* = 578)	Subclavian vein access (*N* = 408)	Total (*N* = 986)	*p*-value
Procedural success, *N* (%)				
Primary access	535 (93)	400 (98)	935 (95)	< 0.001
Secondary access (if primary failed)				
Fluoroscopic axillary vein	15 (31)	2 (29)	17 (30)	
Transcutaneous US-axillary vein	1 (2.0)†	2 (29)‡	3 (5.4)	
Subclavian vein	23 (47)	0 (0)	23 (41)	
Cephalic vein	10 (20)	10 (20)	13 (23)	
Change of access from left to right patient side during procedure	2 (0.3)	2 (0.5)	4 (0.4)	> 0.999
Procedure duration [min]	70 (54, 98)	62 (49, 81)	67 (52, 91)	< 0.001
Fluoroscopy time [min]	5 (3, 10)	5 (3, 9)	5 (3, 10)	0.65
Complications, *N* (%)				
Overall	2 (0.3	17 (4.2)	19 (1.9)	< 0.001
Pocket hematoma requiring revision	0 (0)	2 (0.5)	2 (0.2)	0.171
Hemothorax	0 (0)	3 (0.7)	3 (0.3)	0.071
Pneumothorax requiring drainage	2 (0.3)	8 (2.0)	10 (1.0)	0.020
Pneumothorax (with and without drainage)	2 (0.3)	12 (2.9)	14 (1.4)	0.001

Data are median (IQR) or number (%)

*US* ultrasound

^†^Change from left to right pectoral side during procedure via transcutaneous US-guided transaxillary vein access due to persistent left superior vena cava

^‡^Change from left to right pectoral side during procedure due to access failure

### Success rates of axillary versus subclavian vein access

Axillary access was *successful* in 535/578 patients (93%). The success rates were higher in patients undergoing subclavian vein access with 400/408 (98%) successful implantations (*p* < 0.001) (Fig. 2, Table 2 and Graphical Abstract). Considering only device upgrades or revisions, axillary vein puncture was successful in 69/79 patients (87%). Subclavian vein access succeeded in 45/47 patients (96%) (*p* = 0.208) undergoing device upgrade or revision. Experienced operators (> 300 implants prior to the study period) had lower success rates (91% for new implantations) compared to less experienced operators (95–96%) (Supplement Table S6).

### Complications of axillary versus subclavian vein access

Overall complications occurred in 2/578 patients (0.3%) undergoing axillary vein puncture (Fig. 3, Table 2 and Graphical Abstract). Those complications consisted of pneumothorax requiring drainage in two patients. The complications were more frequent in patients implanted via subclavian vein access with a rate of 17/408 patients (4.2%) (*p* < 0.001): in 12 patients, pneumothorax (2.9%) occurred of which eight required intervention (2%). Three patients suffered from hemothorax and two patients from pocket hematoma necessitating revision (0.5%) (Table 2 and Supplement Table S3). Most complications in patients implanted via the subclavian vein occurred in those treated by less experienced operators (21–50 pacemaker implantations prior to the study period). In contrast, the two complications observed in the axillary group involved patients operated on by more experienced operators (51–300 pacemaker implantations prior to the study period) (Supplement Figure S2).

### Learning curve of US-guided transcutaneous vein access

The learning curves demonstrate a steep rise of successful axillary vein access for the first 25 implantations (Fig. 5). The success rate during the first 10 procedures per operator was 51/60 (85%) (Supplement Table S4). After more than 50 cases per operator the success rate increased to 207/220 (94%).

### Independent predictors of US-guided axillary vein access failure for new implantations

In univariate logistic regression adjusted for the operator, diabetes, increasing left ventricular ejection fraction and the absence of heart failure with reduced ejection fraction were identified as predictors of an US-guided axillary vein access failure in newly implanted patients (Supplement Figure S3). Body Mass Index was not associated with US-guided axillary vein access failure. COPD, diabetes and increasing left ventricular ejection fraction were identified as independent predictors in multivariate logistic regression adjusted for the operator (Supplement Table S5).

## Discussion

Implementation of US-guided axillary venous access was effective and reduced complication rates, mainly by avoiding most pneumothoraxes, in almost 1000 consecutive patients undergoing CIED implantation in a large tertiary care center across a range of experienced and less experienced operators. Feasibility can be underpinned by a steep learning curve resulting in successful axillary vein access in > 90% of patients after 15 procedures for each operator. Not only new implantations but also revisions and upgrades of CIEDs were performed via the axillary vein with high success rates. These data encourage to switch to a default US-guided axillary vein access for implantation or revision and upgrades of devices requiring intracardiac leads.

### Implementing US-guided axillary vein access for device implantations is feasible

**Fig. 4:**
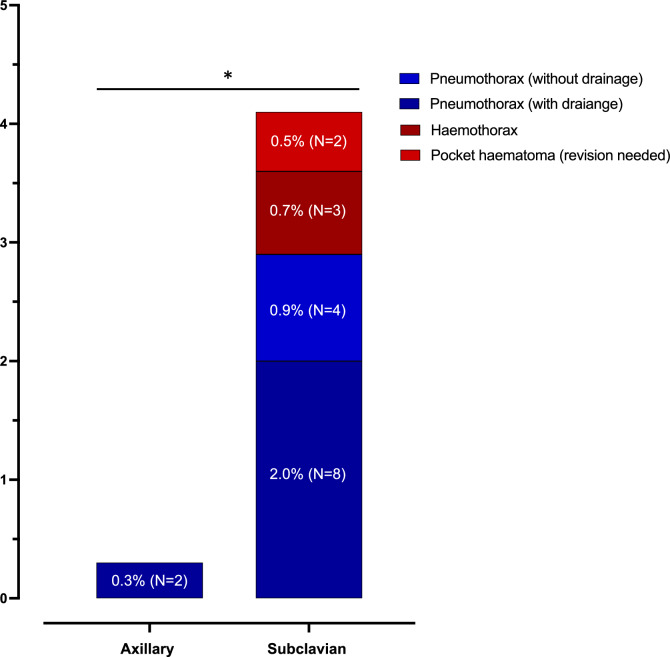
Periprocedural access-related complications stratified by venous access

The success rate of the self-taught, US-guided axillary access for CIED lead implantations was uniformly high (93%). Implanters’ experience ranged from beginners to very experienced. These data replicate success rates reported in smaller series from experienced operators who developed and initially evaluated axillary access (93–94%) [18, 19]. We found a steep learning curve with > 90% successful axillary accesses after 15 procedures per operator (Fig. 4). The success rates reported here are slightly lower than the success rates from a small randomized controlled trial comparing transcutaneous US-guided axillary vein access with cephalic vein access including 88 patients (97.7% after a pre-study training phase) and compared to data from a prospective registry (96.2%) excluding revisions and upgrades and using an out-of-plane US method instead of the in-plane method used in this study [22, 23]. An intra-pocket US-guided access of the axillary vein yielded a success rate of 100% after a pre-study training period in the ACCESS trial [14]. These small differences reflect that our study captured overall success rates from the first US-guided axillary puncture and across a range of operators with variable experience. Interestingly, the success rates were lower among highly experienced operators compared to less experienced ones. This finding may possibly be attributed to the greater familiarity of younger, less experienced operators with US-guided techniques, such as those routinely used for central venous catheter placement. In opposite to the axillary access, the cephalic vein approach is limited by success rates of only 64–86% and by potential difficulties in placing more than two leads [3, 4, 14, 24]. Axillary access failure occurred more frequently in patients without HFrEF and increasing LVEF. These findings are likely explained by decreased central venous pressure. Valsalva maneuver during implantation can help overcome this problem but was not explicitly evaluated in our study. [25] Access failure was also more common in patients with COPD and diabetes in our study. In view of the elevated risk, and the more severe clinical course of pneumothorax in patients with COPD and diabetes, a primary axillary access remains preferable in these patients in our view [26].

In our study, the axillary vein access was associated with a longer procedure time compared to subclavian access. This may be attributed to the recent introduction of the method and the additional steps required for equipment setup, real-time image acquisition, and precise needle guidance. Notably, in the US group, the procedure time began with placement of the probe on the patient’s body, whereas in the subclavian group, timing started with the skin incision; thus, the time required for local anesthesia to take effect—administered prior to the skin incision—would need to be accounted for to ensure a direct comparison. In other studies, after implementing this technique, procedure time for US-guided axillary vein access was shorter compared to fluoroscopy-guided access. [27] In addition, we saw a decrease in procedure time over the study period taking into account additional time for implementing this new approach (Supplemental figure S3).

### Introducing US-guided axillary vein access for device implantations is safe

**Fig. 5:**
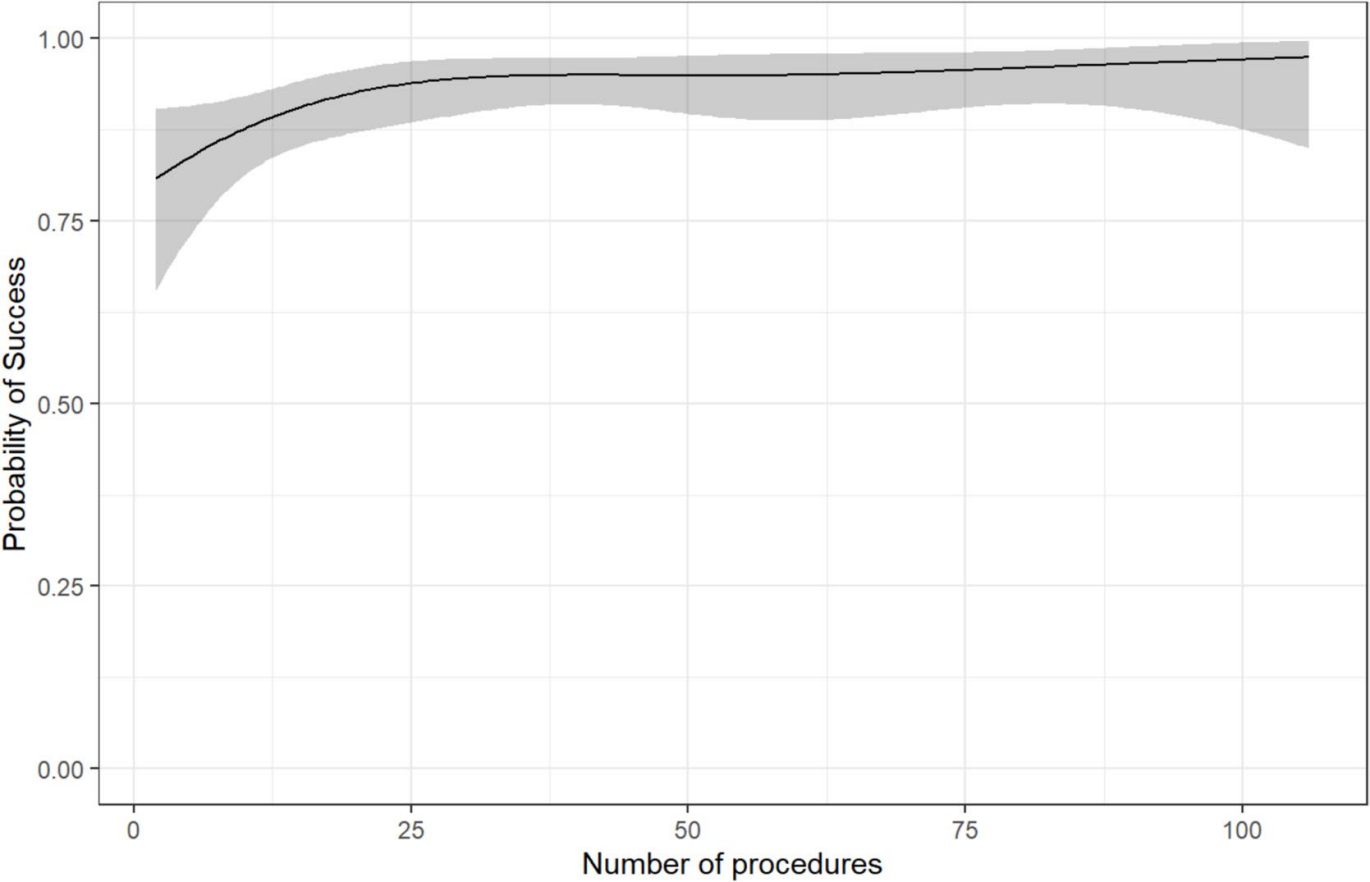
Learning curves for the success of new implantations with transcutaneous US-guided axillary vein access based on the number of procedures performed by operators with at least 30 procedures for all implantations (new implantations and revisions/upgrades)

Introducing US-guided axillary venous access was safe from the first procedure (0.3% complication rate, Table 2 and Fig. 5). Notably, the complications were absent even among less experienced operators. This finding emphasizes the favorable safety profile of the transcutaneous US-guided axillary approach. The complications consisted of two cases of pneumothorax. One of these pneumothoraxes occurred during subclavian puncture during which air was aspirated after unsuccessful axillary attempts. Both complications occurred in patients treated by experienced operators. It is possible that their reliance on ingrained techniques from landmark-guided punctures led to advancing the needle without adequate US visualization, contributing to the occurrence of a pneumothorax. Other, smaller series and small randomized trials did not report any patients with pneumothorax or hemothorax after axillary puncture in series of < 100 patients each, in line with this much larger report (578 patients undergoing axillary vein access). [18, 19, 28] Interestingly, the ZEROFLUOROAXI trial reported two cases of pneumothorax in the US arm (1.5%) [16]. However, as there was a high rate of cross-over to cephalic or fluoroscopic axillary vein access, there is no report on the actual method used before the occurrence of pneumothorax. In the aforementioned ACCESS trial, there were no major pocket hematoma, pneumothorax or hemothorax after intra-pocket US-guided axillary vein access [14]. However, one pocket infection had occurred [14]. Conceptually, there could be an increased risk of CIED and pocket infections due to the intra-pocket US requiring gel in the wound [14]. Although our study was not able to assess the incidence of pocket infections, risk may not be increased with our transcutaneous technique, as the US probe is placed on the skin and not in the wound and only guidewires and not the final leads are passed through the skin. This approach might potentially minimize lead contamination. In addition, the transcutaneous approach allows for an uncomplicated change of laterality without creating unnecessary wounds in case of obstructed access or persistent upper left vena cava. A persistent superior left vena cava occurs in approximately 0.5% of the general population [29]. Accordingly, a change of access sides without leaving a wound on the initial side was performed twice in our axillary access study population.

The rate of 2.0% of pneumothoraxes requiring intervention in our study in patients undergoing subclavian access is in line with results from other reports of 1.2% [5] and 2.4% [6].

With regard to lead long-term lead durability, available data suggest that axillary vein access is associated with longer lead longevity compared with both subclavian access and even cephalic cut down supporting a long-term benefit of the axillary vein access [30].

### US-guided axillary vein access is feasible and safe in CIED revisions and upgrades

US-guided axillary access was used in 79 CIED revision or upgrade procedures without complications in this series. Previous reports on US-guided axillary access included only a small number (14 patients max) of device upgrade procedures [12, 16, 31]. This report provides the first larger data set demonstrating feasibility and safety of US-guided axillary access for device revision and upgrades. This procedure appears attractive for lead revisions or additions as the cephalic vein is iatrogenically occluded if it was chosen for the primary implantation. The success rate of 87% without major complications underpins the feasibility of the US-guided axillary vein approach in patients undergoing CIED revisions or upgrades. However, the success rate was lower compared to new implantations possibly due to limited visualization of the axillary vein because of generator or lead interaction. Maybe an intra-pocket US approach in patients with limited visualization of the axillary vein might be an alternative.

### Strengths and limitations

A strength of this study is the consecutive capture of all patients operated in our center from the first introduction of US-guided axillary access and complete capture of intra-hospital complications. Another strength is the inclusion of all types of CIED procedures requiring venous access, except for lead extractions. A third strength is the protocolized introduction of the new access technique, in a “wedge-type” design and involvement of operators with a wide range of experience in CIED implantations at the time of the study. The comparison was non-randomized and the operators were not blind to the access site. The selection of patients for one or the other access site may have introduced confounders. The time-dependent gradual switch of most operators to axillary access during the study period reduces this bias. The outcomes were determined without blinding to access site. This limitation is mitigated by the fact that complications were determined by ward teams not involved in the procedures and therefore blind to access site. Nonetheless, lack of randomization and lack of blinding remain key limitations of this retrospective study. Generalizability is limited by all-comer data from a single center and the inclusion of patients with heterogeneous device operations. Data on time to venous access and follow-up data exceeding the peri-operative hospital stay were not available. Clearly, future studies with independent validation of our findings and long-term follow-up are needed. Another limitation is the lack of comparison with patients who underwent cephalic vein access.

## Conclusions

In this large-scale study, the implementation of transcutaneous, US-guided access to the axillary vein for implantations, revisions, and upgrades of CIEDs is feasible and can be reliably adopted by inexperienced implanters. Compared to the subclavian vein approach, this method resulted in lower periprocedural access-related complication rates. Larger prospective, randomized trials with longer-follow-up are needed also including patients undergoing revisions and upgrades. The transcutaneous, US-guided axillary vein access might be considered as a primary venous access approach for CIED implantations.

## Supplementary Information

Below is the link to the electronic supplementary material.

Supplementary file1 (DOCX 161 KB)

## Data Availability

The data that support the findings of this study are available from the corresponding author upon reasonable request.

## Abbreviations

CIED: Cardiac implantable electronic devices
CRT: Cardiac resynchronization therapy
ICD: Implantable cardioverter defibrillator
US: Ultrasound
