# Supplementary material for: Implementation of transcutaneous ultrasound-guided axillary vein access for implantations, revisions and upgrades of cardiac implantable electronic devices in a large tertiary care center
Source: Clin Res Cardiol. 2025 Jun 24;115(4):590–601. doi: 10.1007/s00392-025-02692-7 (PMC13013120; doi:10.1007/s00392-025-02692-7)
Supplement: Supplementary file 1 — Supplementary file1 (DOCX 161 KB) [file 392_2025_2692_MOESM1_ESM.docx]

**Supplementary Material**

Supplementary Material to: Nikorowitsch et al., Implementation of transcutaneous ultrasound-guided axillary vein access for implantations, revisions and upgrades of cardiac electronic devices in a large tertiary care centre

**Table of Contents**   **Page number**

**Supplementary Table**

**Supplementary Table S1.** 2

**Supplementary Table S2.** 4

**Supplementary Table S3.** 5

**Supplementary Table S4.** 6

**Supplementary Table S5.** 7

**Supplementary Table S6.** 8

**Supplementary Figure S1.** 9

**Supplementary Figure S2.** 10

**Supplementary Figure S3.** 11

**Supplementary Table S1.** Baseline characteristics according to vein access (transcutaneous US-guided axillary vein access vs. subclavian vein access) for new implantation and revision and upgrades of CIED.

|  | ***New implantation*** | | | ***Revision/upgrade*** | | |
| --- | --- | --- | --- | --- | --- | --- |
|  | **Transcutaneous**  **US-guided transaxillary vein access**  **(N=499)** | **Subclavian vein access**  **(N=361)** | **p-value** | **Transcutaneous**  **US-guided transaxillary vein access**  **(N=79)** | **Subclavian vein access**  **(N=47)** | **p-value** |
| ***Demographic characteristics*** |  |  |  |  |  |  |
| Age [years] (IQR) | 74 (64, 83) | 75 (66, 83) | 0.269 | 71 (60, 82) | 73 (61, 79) | 0.653 |
| Women, N (%) | 192 (38) | 113 (31) | 0.030 | 24 (30) | 20 (43) | 0.166 |
| BMI [kg/m^2^] | 26.0 (23.1, 30.0) | 26.1 (23.2, 29.4) | 0.94 | 26.9 (24.2, 30.0) | 24.8 (23.6, 27.9) | 0.137 |
| ***Comorbidities, N (%)*** |  |  |  |  |  |  |
| Hypertension | 303 (61) | 231 (64) | 0.330 | 43 (54) | 27 (57) | 0.742 |
| Diabetes | 101 (20) | 97 (27) | 0.023 | 16 (20) | 11 (23) | 0.677 |
| Current and prior smoking | 114 (23) | 91 (25) | 0.422 | 18 (23) | 14 (30) | 0.383 |
| Coronary artery disease | 214 (43) | 187 (52) | 0.010 | 36 (46) | 24 (51) | 0.550 |
| Prior thoracic sugery | 14 (2.8) | 23 (6.4) | 0.011 | 2 (2.5) | 4 (8.5) | 0.195 |
| HFrEF | 153 (31) | 99 (28) |  | 49 (64) | 24 (51) |  |
| HFmREF | 48 (9.7) | 41 (11) |  | 7 (9.2) | 8 (17) |  |
| HFpEF | 27 (5.5) | 19 (5.3) |  | 1 (1.3) | 2 (4.3) |  |
| LVEF [%] | 54 (35, 60) | 55 (39, 60) | 0.26 | 35 (25, 50) | 40 (30, 57) | 0.12 |
| Pulmonary disease, N (%) | 73 (15) | 40 (11) | 0.13 | 10 (13) | 6 (13) | 0.99 |
| COPD, N (%) | 40 (8.0) | 17 (4.7) | 0.05 | 5 (6.3) | 4 (8.5) | 0.73 |
| ***Antiplatelet therapy/anticoagulation, N (%)*** |  |  | 0.004 |  |  | 0.89 |
| No | 297 (60) | 184 (51) |  | 59 (75) | 34 (72) |  |
| ASS | 116 (23) | 98 (27) |  | 14 (18) | 8 (17) |  |
| Clopidogrel | 50 (10) | 29 (8.0) |  | 4 (5.1) | 4 (8.5) |  |
| DAPT | 35 (7.0) | 47 (13) |  | 2 (2.5) | 1 (2.1) |  |
| Ticagrelor/Prasugrel | 1 (0.2) | 3 (0.8) |  | 0 (0) | 0 (0) |  |
| ***Device indication, N (%)*** |  |  |  |  |  |  |
| AV block | 211 (42) | 226 (63) |  | 37 (47) | 21 (45) |  |
| Sinus node dysfunction |  |  |  |  |  |  |
| Binodal disease | 0 (0) | 1 (0.3) |  | 0 (0) | 0 (0) |  |
| Slow AF | 36 (7.2) | 25 (6.9) |  | 2 (2.5) | 4 (8.5) |  |
| Resynchronization therapy | 36 (7.2) | 10 (2.8) |  | 9 (11) | 6 (13) |  |
| ICD for primary prevention | 76 (15) | 23 (6.4) |  | 16 (20) | 10 (21) |  |
| ICD for secondary prevention | 85 (17) | 43 (12) |  | 31 (39) | 11 (23) |  |
| ***Type of device, N (%)*** |  |  | <0.001 |  |  | 0.436 |
| Single chamber PM | 11 (14) | 32 (8.9) |  | 1 (1.3) | 1 (2.1) |  |
| Dual chamber PM | 271 (54) | 256 (71) |  | 23 (29) | 21 (45) |  |
| Single chamber ICD | 87 (17) | 39 (11) |  | 10 (13) | 3 (6.4) |  |
| Dual chamber ICD | 30 (6.0) | 16 (4.4) |  | 8 (10) | 6 (13) |  |
| CRT-P | 20 (4.0 | 7 (1.9) |  | 11 (14) | 5 (11) |  |
| CRT-D | 41 (8.2) | 11 (3.0) |  | 26 (33) | 11 (23) |  |
| **Number of new leads, N (%)** | 2 (1, 2) | 2 (2, 2) | 0.67 | 1 (1, 1) | 1 (1, 2) | 0.63 |
| 0 (reuse old lead) | - | - |  | 2 (2.5) | 1 (2.1) |  |
| 1 | 138 (28) | 72 (20) |  | 59 (75) | 33 (70) |  |
| 2 | 303 (61) | 274 (76) |  | 15 (19) | 13 (28) |  |
| 3 | 58 (12) | 15 (4.2) |  | 3 (3.8) | 0 (0) |  |

Data are median (IQR) or number (%).

Abbreviations: AF, atrial fibrillation; ASS; acetylsalicylic acid, BMI, body mass index; DAPT, dual antiplatelet therapy; CIED, cardiac implantable electronic devices; COPD, chronic obstructive pulmonary disease; CRT-D/-P, cardiac resynchronization therapy defibrillator or pacemaker; LVEF, left ventricular ejection fraction; HFmreF, heart failure with mildly reduced ejection fraction; HFpEF, heart failure with preserved ejection fraction; HFreF, heart failure with reduced ejection fraction; ICD, implantable cardioverter defibrillator; US, ultrasound; PM, pacemaker

**Supplementary Table S2.** Number of procedures per operator stratified according to the maximum of operations performed during the study period.

|  | **Maximum number of procedures per operator, N** | | | |
| --- | --- | --- | --- | --- |
|  | **1-10** | **11-30** | **31-50** | **>50** |
| **Number of new implantations or revision/upgrade in the study, N** | 67 | 130 | 197 | 592 |
| **Number of operators, N** | 17 | 7 | 5 | 4 |
| **Initially chosen access site** |  |  |  |  |
| Transcutaneous US-guided axillary vein access, N (%) | 15 (22) | 46 (35) | 97 (49) | 420 (71) |
| Subclavian vein access, N (%) | 52 (78) | 84 (65) | 100 (51) | 172 (29) |

**Table S3.** Outcomes of the study. Procedural success and perioperative complications by venous access group (transcutaneous US-guided axillary vein access vs. subclavian vein access) for new implantation and revision and upgrades of CIED

|  | ***New implantation*** | | | ***Revision/upgrade*** | | |
| --- | --- | --- | --- | --- | --- | --- |
|  | **Transcutaneous**  **US-guided transaxillary vein access**  **(N=499)** | **Subclavian vein access**  **(N=361)** | **p-value** | **Transcutaneous**  **US-guided transaxillary vein access**  **(N=79)** | **Subclavian vein access**  **(N=47)** | **p-value** |
| ***Procedural success, N (%)*** |  |  |  |  |  |  |
| Primary access | 466 (93) | 355 (98) | <0.001 | 69 (87) | 45 (96) | 0.208 |
| Secondary access (if primary failed) |  |  |  |  |  |  |
| *Fluoroscopic axillary vein* | 10 (27) | 1 (20) |  | 5 (42) | 1 (50) |  |
| *Transcutaneous US-axillary vein* | 1 (2.7) | 1 (20) |  | 0 (0) | 1 (50) |  |
| *Subclavian vein* | 17 (46) | 0 (0) |  | 6 (50) | 0 (0) |  |
| *Cephalic vein* | 9 (24) | 3 (60) |  | 1 (8.3) | 0 (0 |  |
| *Change of laterality from left to right patient side during procedure* | 2 (0.4) | 2 (0.6) | >0.999 | 0 (0) | 0 (0) |  |
| ***Procedure*** |  |  |  |  |  |  |
| Duration [min] | 68 (53, 90) | 61 (48, 78) | <0.001 | 100 (70, 128) | 80 (54, 122) | 0.06 |
| Fluroscopy time [min] | 5 (3, 9) | 5 (3, 9) | 0.39 | 7 (3, 16) | 7 (3, 13) | 0.47 |
| ***Periprocedural complications, N (%)*** |  |  |  |  |  |  |
| *Overall* | 2 (0.4) | 16 (4.4) | <0.001 | 0 (0) | 1 (2.1) | 0.373 |
| Pocket haematoma requiring revision | 0 (0) | 2 (0.6) | 0.176 | 0 (0) | 0 (0) |  |
| Haemothorax | 0 (0) | 0 (0) | 0.176 | 0 (0) | 1 (2.1) | 0.373 |
| Pneumothorax  *requiring drainage* | 2 (0.4) | 8 (2.2) | 0.021 | 0 (0) | 0 (0) |  |
| Pneumothorax  *(with and without drainage)* | 2 (0.4) | 12 (3.3) | <0.001 | 0 (0) | 0 (0) |  |

Abbreviations: CIED, cardiac implantable electronic devices; US, ultrasound

**Supplementary Table S4.** Procedural success and perioperative complications for US-guided axillary vein access stratified according to the increasing number of procedures per operator for operators with >30 procedures.

|  | **1-10,**  **N = 60** | **11-30,**  **N = 120** | **31-50,**  **N = 95** | **>50,**  **N = 220** | **p-value** |
| --- | --- | --- | --- | --- | --- |
| ***Typ of Procedure*** |  |  |  |  |  |
| *New implantation* | 56 (93) | 104 (87) | 76 (80) | 190 (86) |  |
| *Revision or upgrade* | 4 (6.7) | 16 (13) | 19 (20) | 30 (14) |  |
| **Success first puncture site axillary vein** | 51 (85) | 111 (93) | 86 (91) | 207 (94) | 0.14 |
| **Duration [min]** | 79 (61, 117) | 70 (54, 100) | 69 (54, 90) | 68 (51, 107) | 0.15 |
| **Fluroscopy time [min]** | 6 (3, 13) | 4 (3, 10) | 5 (3, 9) | 5 (3, 12) | 0.8 |
| ***Periprocedural complications, N (%)*** |  |  |  |  |  |
| *Overall* | 0 (0) | 1 (0.8) | 0 (0) | 0 (0) | >0.9 |
| Pocket haematoma requiring revision | 0 (0) | 0 (0) | 0 (0) | 0 (0) | 0 (0) |
| Haemothorax | 0 (0) | 0 (0) | 0 (0) | 0 (0) | 0 (0) |
| Pneumothorax  *requiring drainage* | 0 (0) | 1 (0.8) | 0 (0) | 1 (0.5) | >0.9 |
| Pneumothorax  *(with and without drainage)* | 0 (0) | 1 (0.8) | 0 (0) | 1 (0.5) | >0.9 |

**Supplementary Table S5.** Multivariate logistic regression model for US-guided axillary vein puncture failure for new implantations. Adjusted for significant predictors from univariate model and adjusted for all operators with min 30 new implantations.

|  | **OR** | **95% CI** | **p-value** |
| --- | --- | --- | --- |
| **COPD** | 7.29 | 2.0-24.6 | 0.002 |
| **Diabetes** | 5.65 | 2.2-14.5 | <0.001 |
| **LVEF** | 4.21 | 2.24-8.94 | <0.001 |

Abbreviations: COPD, chronic obstructive pulmonary disease: CI, confidence interval; LVEF, left ventricular ejection fraction; OR, odds ratio

**Supplementary Table S6.** Procedural success stratified according to the number of CIED implantations performed by each operator before the study period considering only new implantations.

| **Number of Procedures before study period** | **0-20 N = 62** | **21-50 N = 72** | **51-300 N = 382** | **>300**  **N = 344** |
| --- | --- | --- | --- | --- |
| **Primary puncture site** |  |  |  |  |
| *Axillary vein ultrasound* | 20 (32%) | 45 (63%) | 221 (58%) | 213 (62%) |
| *Subclavian vein* | 42 (68%) | 27 (38%) | 161 (42%) | 131 (38%) |
| Success first puncture site | 59 (95%) | 70 (97%) | 372 (97%) | 320 (93%) |
| Success first puncture site axillary vein | 19 (95%) | 43 (96%) | 211 (95%) | 193 (91%) |
| Success first puncture site subclavian vein | 40 (95%) | 27 (100%) | 161 (100%) | 127 (97%) |

**Supplementary Figure S1.** Exploratory analysis of the association between duration of procedure and date of new implantation of DDD pacemaker with axillary vein access as primary puncture site, using LOESS plots.


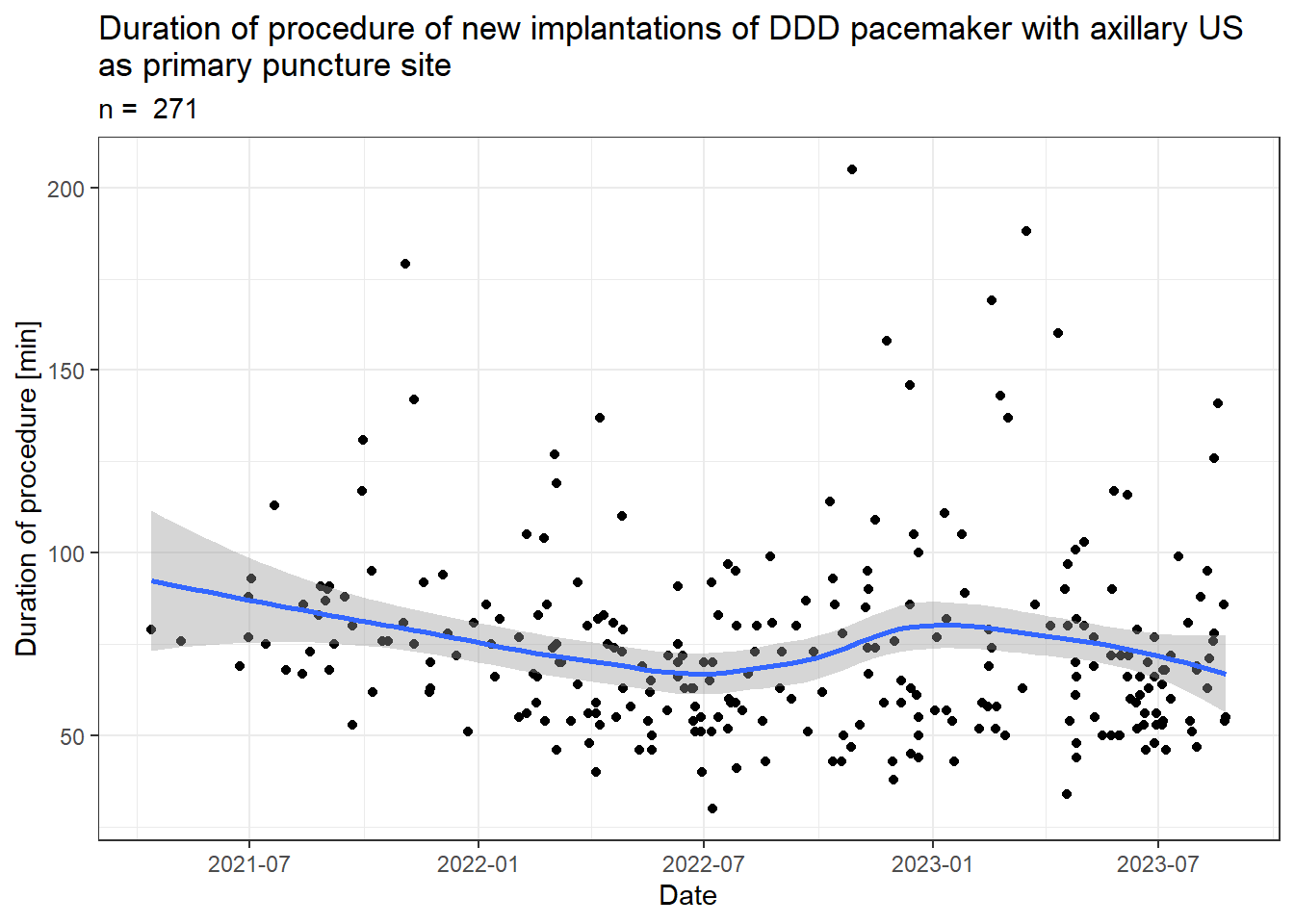


**Supplementary Figure S2.** Complications stratified according to access site and according to operator experience. The numbers on the x axis displayed represent the number of pacemaker implantations performed by each operator before the study period.


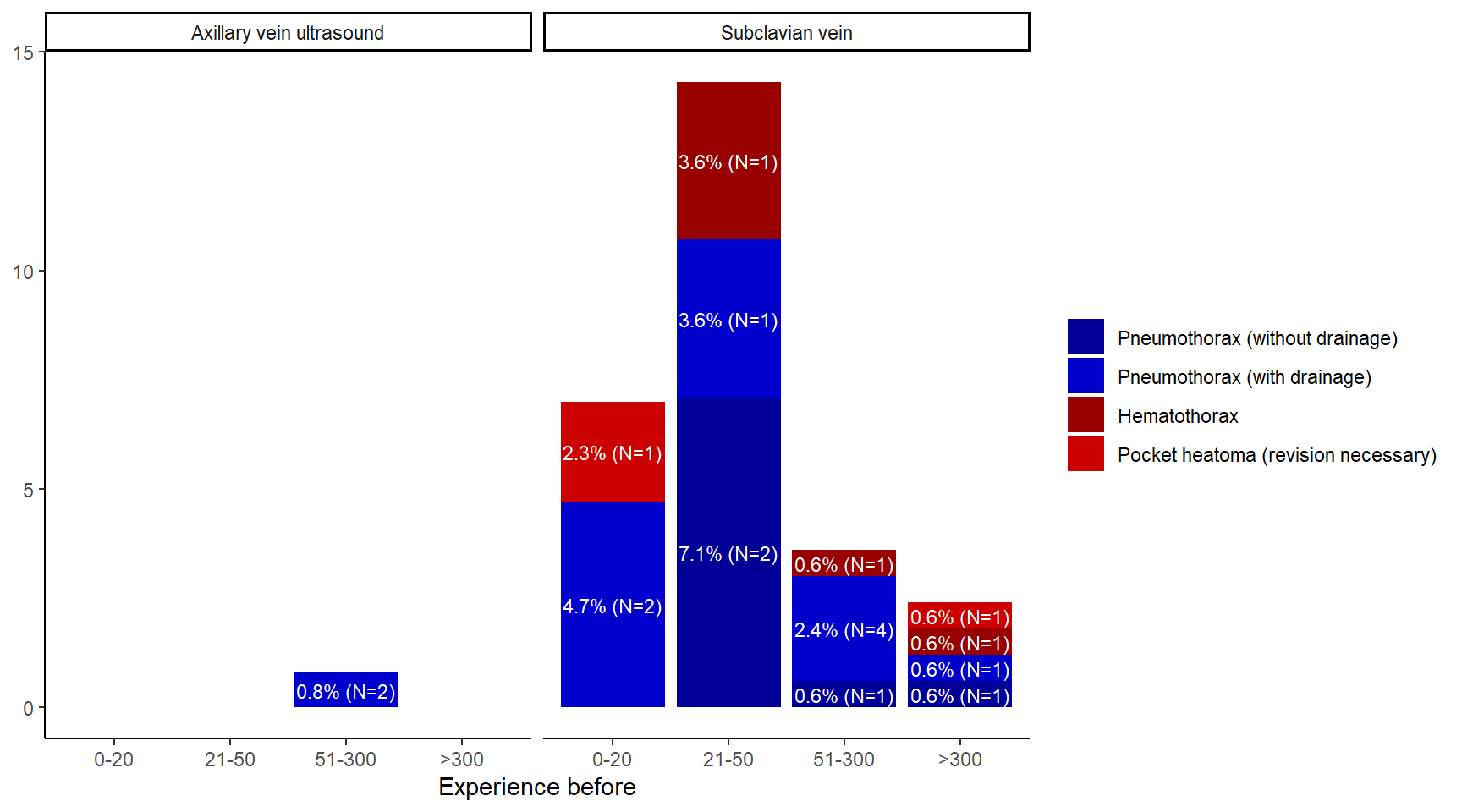


**Supplementary Figure S3.** Univariate logistic regression model US-guided axillary vein puncture failure for new implantation with adjustment for all operators with min 30 new implantations.

Abbreviations: BMI, body mass index; COPD, chronic obstructive pulmonary disease; LVEF, left ventricular ejection fraction; HFmreF, heart failure with mildly reduced ejection fraction; HFpEF, heart failure with preserved ejection fraction; HFreF, heart failure with reduced ejection fraction; ICD, implantable cardioverter defibrillator; US, ultrasound
